# Prognostic significance of serum aspartic transaminase in diffuse large B-cell lymphoma

**DOI:** 10.1186/s12885-019-5758-2

**Published:** 2019-06-08

**Authors:** Ting-Xun Lu, Shuang Wu, Dong-Yan Cai, Ting-Ting Hong, Ying Zhang, Hua-Qiang Gao, Hai-Ying Hua, Xiao-Hong Wu

**Affiliations:** 1 Department of Oncology, Affiliated Hospital of Jiangnan University, Wuxi, 214000 Jiangsu People’s Republic of China; 2 Department of Hematology, Affiliated Hospital of Jiangnan University, Wuxi, 214000 People’s Republic of China; 3 Department of Hematology, The Third Affiliated Hospital of Nantong University, The Third People’s Hospital of Wuxi, Wuxi, 214000 People’s Republic of China

**Keywords:** Non-Hodgkin lymphoma, Diffuse large B-cell lymphoma, Aspartic transaminase, Prognosis, Survival

## Abstract

**Background:**

Liver function is routinely assessed in clinical practice as liver function tests provide sensitive indicators of hepatocellular injury. However, the prognostic value of enzymes that indicate hepatic injury has never been systematically investigated in lymphoma, including diffuse large B-cell lymphoma (DLBCL).

**Methods:**

This study examined the prognostic value of baseline aspartic transaminase (AST) in DLBCL patients. The association between AST and clinical features was analyzed in 179 DLBCL patients treated from 2006 to 2016. All enrolled patients were treated with R-CHOP or R-CHOP-like chemotherapy. Log-rank test, univariable analysis, and subgroup analysis were performed to evaluate the impact of AST on survival.

**Results:**

AST 33.3 U/L was considered to be the optimal threshold value for predicting prognosis. A higher AST level was associated with advanced stage (*P* = 0.001), poorer performance status (*P* = 0.014), elevated lactate dehydrogenase level (*P* <  0.0001), presence of B symptoms (*P* = 0.001), high-risk International Prognostic Index (IPI, IPI 3–5) (*P* = 0.002), non-germinal center B-cell subtypes (*P* = 0.038), hepatitis B virus surface antigen positivity (*P* = 0.045) and more extra nodal involvement (ENI, ENI ≥ 2) (*P* = 0.027). Patients with a higher AST level had a shorter overall survival (OS) (2-year OS rate, 53.6% vs. 83.6%, *P* <  0.001). Subgroup analysis indicated that higher AST levels have poorer prognostic values in patients without B symptoms and LDH positive groups.

**Conclusion:**

A pretreatment AST level is associated with OS in DLBCL patients treated with R-CHOP or similar chemotherapy regimens. A high pretreatment AST level might be a reliable prognostic factor for predicting a dismal outcome in DLBCL patients. Serum AST levels may be investigated for use as an easily determinable, inexpensive biomarker for risk assessment in patients with DLBCL.

**Electronic supplementary material:**

The online version of this article (10.1186/s12885-019-5758-2) contains supplementary material, which is available to authorized users.

## Background

Diffuse large B-cell lymphoma (DLBCL) is one of the most common forms of malignant lymphoma, accounting for 30–40% of all newly diagnosed cases of adult non-Hodgkin lymphoma (NHL). DLBCL is a heterogeneous disease, in terms of morphology, clinical features, biological behavior, and response to therapies [1], which makes predicting patient prognosis challenging. The International Prognostic Index (IPI) and the gene expression profile classification are the most widely accepted prognostic scoring systems. However, there are still patients with a favorable IPI who suffer from disease recurrence [2, 3], and gene expression profiling is not yet a practical prognostic tool in routine practice.

Molecular markers and immunohistochemical profiling are other prognostic strategies in DLBCL currently in use, but these methods are sometimes costly and always rely on tissue samples. Therefore, alternative readily available prognostic biomarkers with low clinical costs are urgently needed to improve risk assessment in DLBCL patients.

Liver function is routinely assessed in clinical practice as liver function tests provide sensitive indicators of hepatocellular injury. Importantly, it has been reported that high aspartate aminotransaminase (AST) levels in breast cancer patients are not always associated with significant liver disease but they do correlate with an advanced clinical stage of breast cancer [4]. Furthermore, a higher preoperative AST/alanine aminotransaminase (ALT) ratio predicts poor outcome in patients with upper tract urothelial cancer [5]. Similarly, high serum AST levels before surgery may serve as a valuable prognostic marker in non-small cell lung cancer (NSCLC) [6]. Thus, we analyzed the clinical significance and the prognostic value of these two enzymes that indicate hepatic injury, ALT or glutamic-pyruvic transaminase and AST or glutamic oxaloacetic transaminase, in patients with de novo DLBCL.

## Methods

### Patient selection

We reviewed medical records of 207 patients who were diagnosed, according to the 2016 World Health Organization classification, with de novo DLBCL at the Affiliated Hospital of Jiangnan University and Affiliated Hospital of Nantong University between 2006 and 2016. Blood samples of all patients were collected at the time of diagnosis or before the beginning of treatment. No patients with relapse after treatment were included in this study. Likewise, patients with primary central nervous system lymphoma, primary mediastinal B-cell lymphoma, post-transplant lymphoproliferative disorders, transformed NHL, and HIV-positive DLBCL were excluded from the study. Further cases also excluded from the study, including patients with a history of hepatitis (9/207, 4.3%), chronic alcohol ingestion (5/207, 2.4%), cirrhosis, recent medication for hepatotoxicity (2/207, 1.0%), and fatty liver (2/207, 1.0%), patients with pretreatment liver function injury due to viral infection (10/207, 4.8%), and patients with concomitant or past liver cancer (none). Consequently, 179 patients with DLBCL qualified for the study. All enrolled patients were treated with R-CHOP (rituximab plus cyclophosphamide, doxorubicin, vincristine, and prednisone) or R-CHOP-like chemotherapy. Because this is a retrospective study, all recruited patients were informed and verbal consent was obtained in accordance with the requirements of the Declaration of Helsinki, and the research project was approved by the review boards of Jiangnan University, Affiliated Hospital of Jiangnan and Nantong Universities.

### Optimal cutoff value by X-tile software

X-tile 3.6.1 software (Yale University, New Haven, CT, USA) was used to determine the optimal cut-off values for ALT and AST. However, the optimal cutoff value for ALT was not found. Thus, we used 50 U/L for ALT according to the reference value in a liver function test. The optimal cutoff value was 33.3 U/L for AST according to the X-tile software recommendation. Results provided by the X-tile software showed that a dichotomy in the AST level had a better prognostic value than a trichotomy (Additional file 1: Figure S1 and Additional file 2: Figure S2).

### Differential gene expression using the gene expression omnibus (GEO) database data

The DLBCL microarray and corresponding clinical data in the present study were downloaded from the public GEO database (http://www.ncbi.nlm.nih.gov/geo/) with the accession number GSE27255. Raw data from the GEO database were extracted using the R Bioconductor limma and impute packages (http://bioconductor.org/biocLite.R). The data were presented as Volcano and Heatmap plots. The selected mRNAs were examined in cell lines of germinal center B-cell (GCB) and activated B-cell (ABC) subtype to explore if any significant differences in expression exist. Using Mann-Whitney U test, results of fold change > 1 and *P* < 0.05 between GCB and ABC cell lines were considered significant.

### Immunohistochemistry

Immunohistochemistry was performed on 4-μm formalin-fixed paraffin-embedded sections. The antibodies used in the study were for CD20 (clone L26, Abcam, cutoff: 30%), CD10 (clone 56C6, Dako, cutoff: 30%), Bcl6 (clone LN22, Dako, cutoff: 30%), MUM1 (clone MUM1p, Dako, cutoff: 30%), and Myc (clone Y69; Abcam, cutoff: 40%). The cutoff values for each antibody have been previously described [7].

### Statistical analysis

Univariable tests were used to compare categorical (Chi-square and Fisher’s exact) and continuous (Student’s t test or Kruskal-Wallis test when appropriated) variables. Survival curves were plotted using the Kaplan–Meier method and were compared using log-rank test. According to Cheson 2014, overall survival (OS) was defined as the time from diagnosis to death; patients who remained alive were censored at the last date of follow-up [8]. Statistical analysis was performed using SPSS software, Version 20.0. For all the tests, *P* < 0.05 (2-sided) was considered statistically significant.

## Results

### Patients’ characteristics

The median age of the cohort was 57 years (range 18–88 years old). The median follow up time was 28 months (3–112 months). The median treatment cycle was 6 cycles (4–8 cycles). The frequencies with elevated ALT and AST levels were 12.3% (22/179) and 24.6% (44/179), respectively. The baseline clinical parameters of the patients are presented in Table 1.

**Table 1:** Clinical characteristics of the 179 DLBCL patients from 2006 to 2016

Characteristics	No. of cases (%)
Age (years)	
≤60	101 (56.4)
Male	104 (58.1)
Stage III-IV	99 (55.3)
Elevated ALT (> 50 U/L)	22 (12.3)
Elevated AST (> 33.3 U/L)	44 (24.6)
Elevated LDH (> 250 U/L)	72 (40.2)
ECOG PS ≥2	28 (15.6)
ENI ≥2	43 (24.0)
IPI score of 3–5	52 (29.1)
B symptoms	62 (34.6)
COO (Hans)	
GCB	77 (43.0)
Treatment	
R-CHOP	145 (81.0%)
R-DA-EPOCH^a^	14 (7.8%)
R-CHOP-like^b^	20 (11.2%)
Radiation^c^	11 (6.1%)
Auto-HSCT consolidation^d^	15 (8.4%)

Abbreviations: *ALT* alanine aminotransferase, *IPI* International Prognostic Index, *AST* aspartate aminotransferase, *COO* cell of origin, *DLBCL* diffuse large B-cell lymphoma, *ECOG PS* performance status of Eastern Cooperative Oncology Group, *ENI* extra nodal involvement, *GCB* germinal-center B-cell type, *HSCT* Hematopoietic Stem Cell Transplantation, *LDH* lactate dehydrogenase

^a^Patients with high risk (IPI = 4–5) received R-DA-EPOCH regimen

^b^Patients with old age (≥ 75 years old), extremely poor ECOG PS or accompanying heart disease received R-CHOP-like regimens including R-CDOP, R-CEOP and R-mini-CHOP

^c^Cases with localized residual lesions received radiotherapy after immunochemotherapy

^d^Among the patients who received Auto-HSCT, 8 cases were AST positive

### mRNA levels of ALT and AST in patients with lymphoma in GEO

**Fig. 1:**
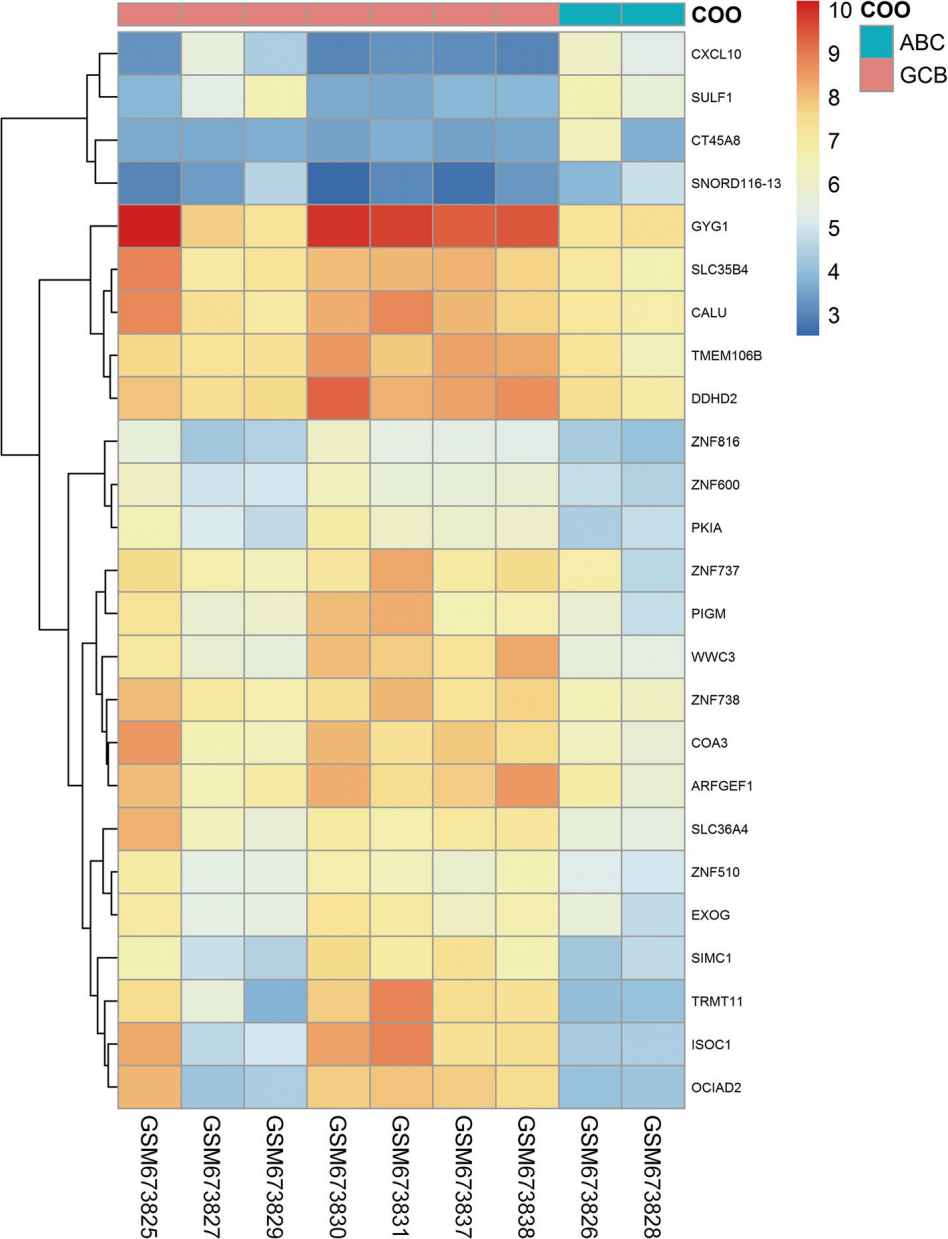
Heatmap plot of different expressed genes with GEO data in human DLBCL cell lines. Heat map hierarchical clustering reveals 25 genes were differentially expressed between GCB and ABC DLBCL cell lines. Neither ALT nor AST was among the different expressed genes. Abbreviations: ALT: alanine aminotransaminase; AST: aspartate aminotransferase; DLBCL: diffuse large B-cell lymphoma; GEO: gene expression omnibus

**Fig. 2:**
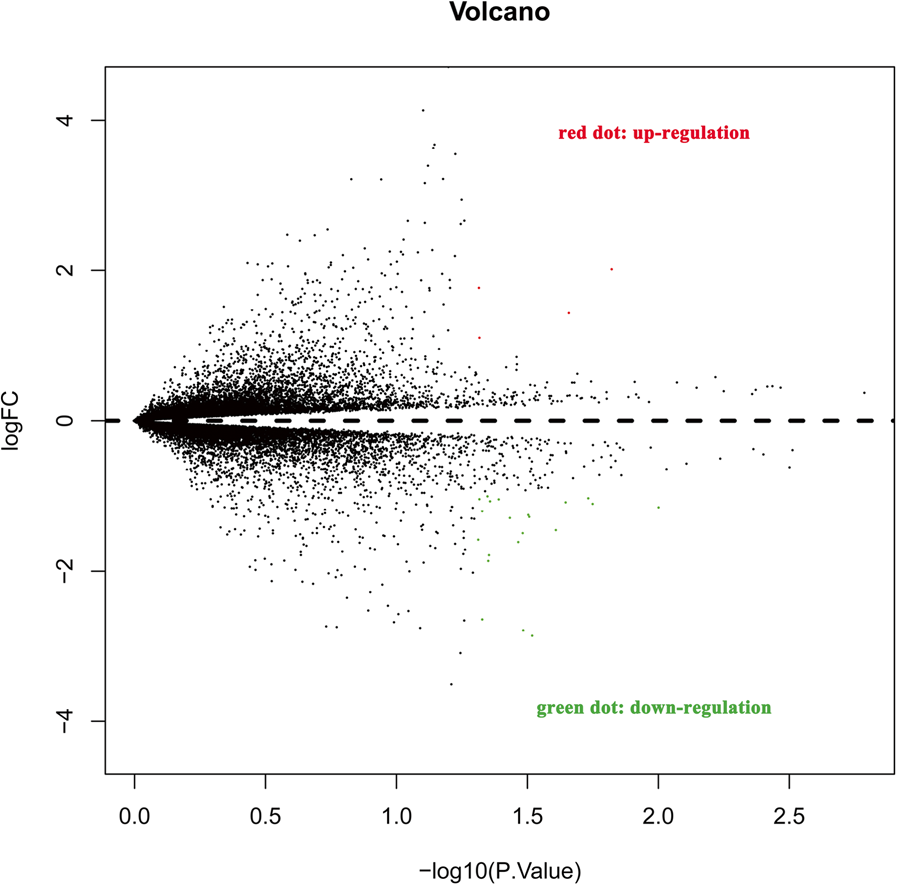
Volcano plot of different expressed genes with GEO data in human DLBCL cell lines. The red dot represents up-regulated genes, and the green dot represents down-regulated genes. Abbreviations: DLBCL: diffuse large B-cell lymphoma; GEO: gene expression omnibus

DLBCL patients with different cells of origin (COO) have distinct outcomes; therefore, we analyzed the differently expressed genes in the GCB and ABC subtypes. We speculated that higher ALT or AST mRNA levels were more frequent in the ABC subgroup. Thus, the Human DLBCL of the Affymetrix Human Gene 1.0 ST Array microarray data were downloaded from the public GEO database. Differently expressed genes in the DLBCL cell lines of the GCB and ABC subtypes (Table 2) were analyzed. A total of 25 genes were differentially expressed as shown in the Volcano and Heatmap plots (Figs. 1 and 2). However, the expression of ALT and AST mRNA was similar between the GCB and ABC cell lines. These results suggest that the mRNA levels of ALT or AST may not be reliant on the COO.

**Table 2:** DLBCL cell lines of GCB and ABC subtype analyzed in this study

Sample	Title	COO
GSM673825	OCI-Ly1	GCB
GSM673826	OCI-Ly3	ABC
GSM673827	OCI-Ly4	GCB
GSM673828	OCI-Ly10	ABC
GSM673829	OCI-Ly18	GCB
GSM673830	OCI-Ly19	GCB
GSM673838	SUDHL6	GCB
GSM673831	SUDHL8	GCB
GSM673837	SUDHL4	GCB

Abbreviations: *ABC* active B-cell type, *COO* cell of origin, *DLBCL* diffuse large B-cell lymphoma, *GCB* germinal-center B-cell type

### Association between clinical features and ALT or AST level

**Fig. 3:**
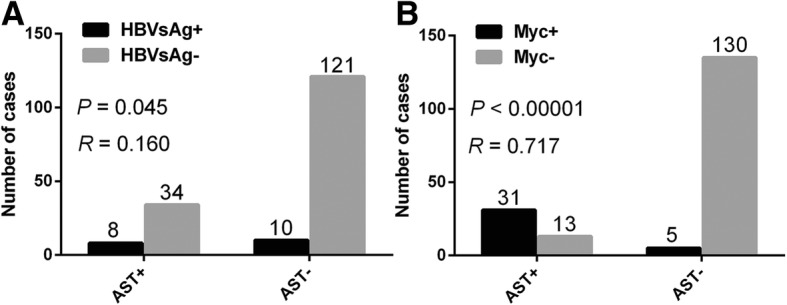
The distribution of HBsAg and Myc protein expression levels with different AST status. The correlations of HBsAg (**a**), Myc protein expression levels (**b**) to AST status. Abbreviations: AST: aspartate aminotransferase; HBsAg: hepatitis B virus surface antigen

In the present cohort, ALT-positive and ALT-negative patients had similar clinical features. However, AST positivity was significantly associated with advanced Ann Arbor stage (stage III or IV) (*P* = 0.001), poor performance status (PS) (*P* = 0.014), elevated lactate dehydrogenase (LDH) level (*P* < 0.0001), more extra nodal involvement (ENI, ENI ≥ 2) (*P* = 0.027), high-risk IPI (IPI 3–5) (*P* = 0.002), presence of B symptoms (*P* = 0.001), hepatitis B virus surface antigen (HBsAg) positivity (*P* = 0.045) (Fig. 3a), Myc protein positivity (*P* < 0.0001) (Fig. 3b), and non-GCB subtypes (*P* = 0.038) (Table 3).

**Table 3:** Relationships between clinical features and ALT and AST status

Characteristics	ALT^+^	ALT^−^	*P* value	AST^+^	AST^−^	*P* value
	No. of cases			No. of cases		
Age (years)						
≤60	16	85	0.100	27	74	0.447
> 60	6	72		17	61	
Sex						
Male	16	88	0.138	24	80	0.582
Female	6	69		20	55	
Stage						
III-IV	12	87	0.939	34	65	0.001
I-II	10	70		10	70	
ECOG PS						
≥ 2	6	22	0.121	12	16	0.014
< 2	16	135		32	119	
LDH						
Over ULN	13	59	0.054	36	36	< 0.0001
Normal	9	98		8	99	
ENI						
≥ 2	5	38	0.250	16	27	0.027
< 2	17	119		28	108	
IPI						
3–5	7	45	0.760	21	31	0.002
0–2	15	112		23	104	
B symptoms						
Positive	11	51	0.106	24	38	0.001
Negative	11	106		20	97	
COO (Hans)						
GCB	6	71	0.111	13	64	0.038
Non-GCB	16	86		31	71	

Abbreviations: *ALT* alanine aminotransferase, *AST* aspartate aminotransferase, *COO* cell of origin, *ECOG PS* performance status of Eastern Cooperative Oncology Group, *ENI* extra nodal involvement, *GCB* germinal-center B-cell type, *LDH* lactate dehydrogenase, *IPI* International Prognostic Index

### Survival analysis associated with ALT and AST levels

**Fig. 4:**
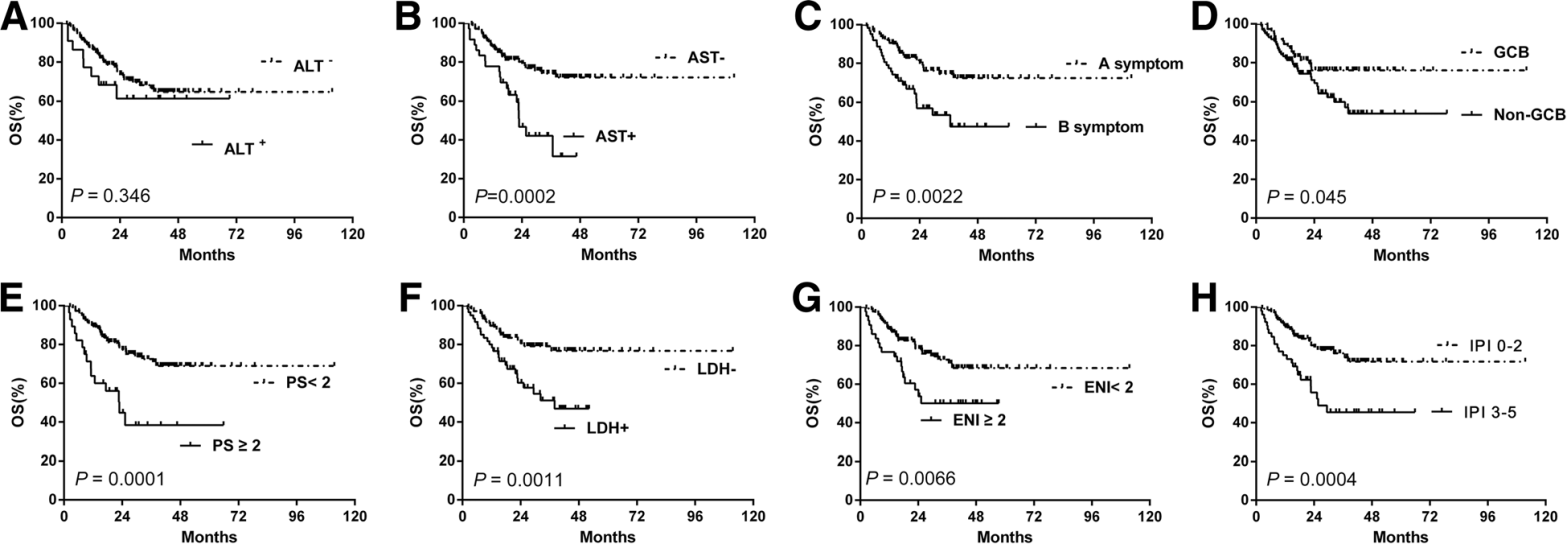
The differences of overall survival in cases grouped according to ALT (**a**), AST (**b**), A or B symptom (**c**), histological type (**d**), PS status (**e**), LDH level (**f**), extranodal involvement (**g**) and IPI risk stratification (**h**). Abbreviations: ALT: alanine aminotransferase; AST: aspartate aminotransferase; ECOG PS: performance status of Eastern Cooperative Oncology Group; ENI: extra nodal involvement; GCB: germinal-center B-cell type; IPI: International Prognostic Index; LDH: lactate dehydrogenase; OS: overall survival

**Fig. 5:**
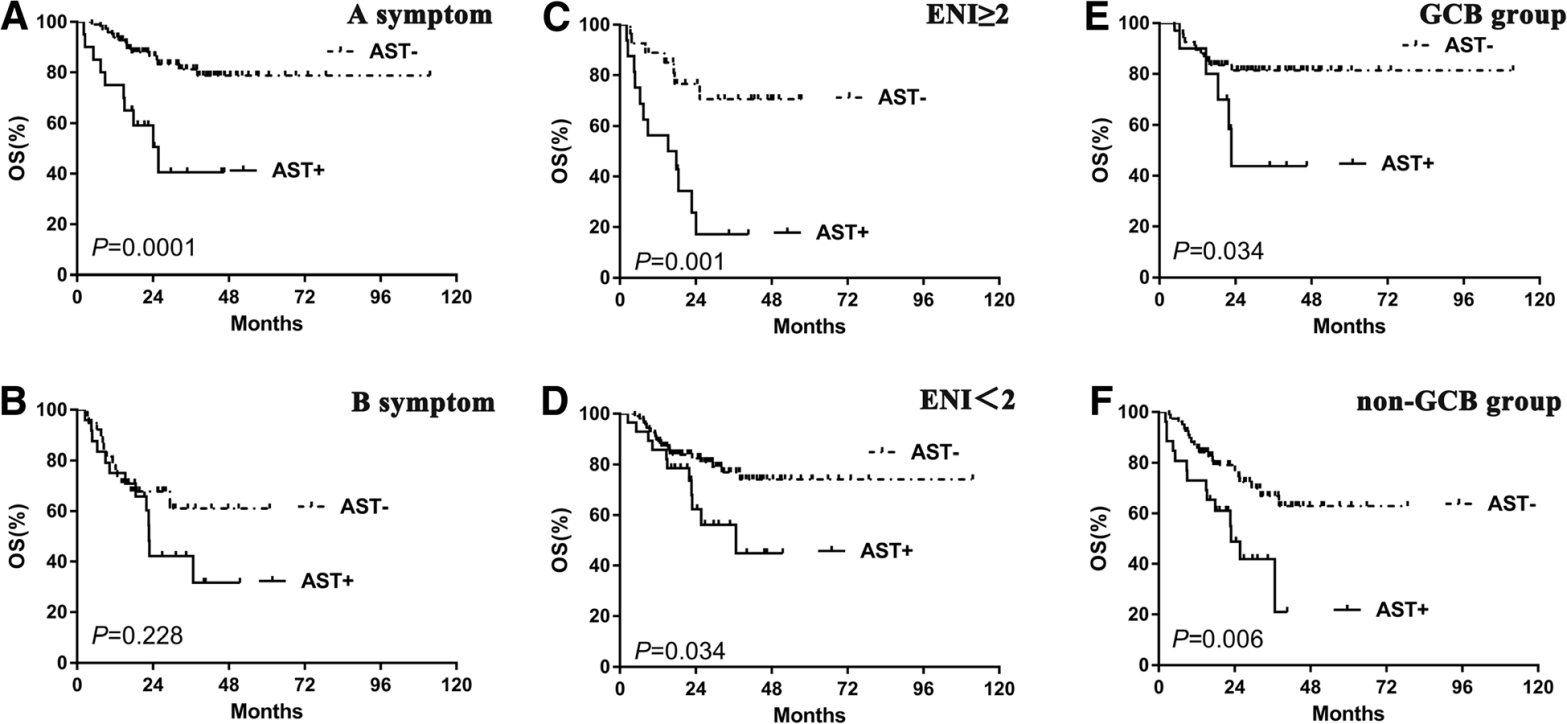
Subgoup analysis with different clinicopathological factors. Patients of higher AST level had superior prognostic value in “A symptoms” group (**a**). Patients of higher AST level showed poorer outcome regardless of ENI status (**c-d**) and COO (**e-f**). Abbreviations: AST: aspartate aminotransferase; COO: cells of origin; ENI: extra nodal involvement; GCB: germinal-center B-cell type; OS: overall survival

**Fig. 6:**
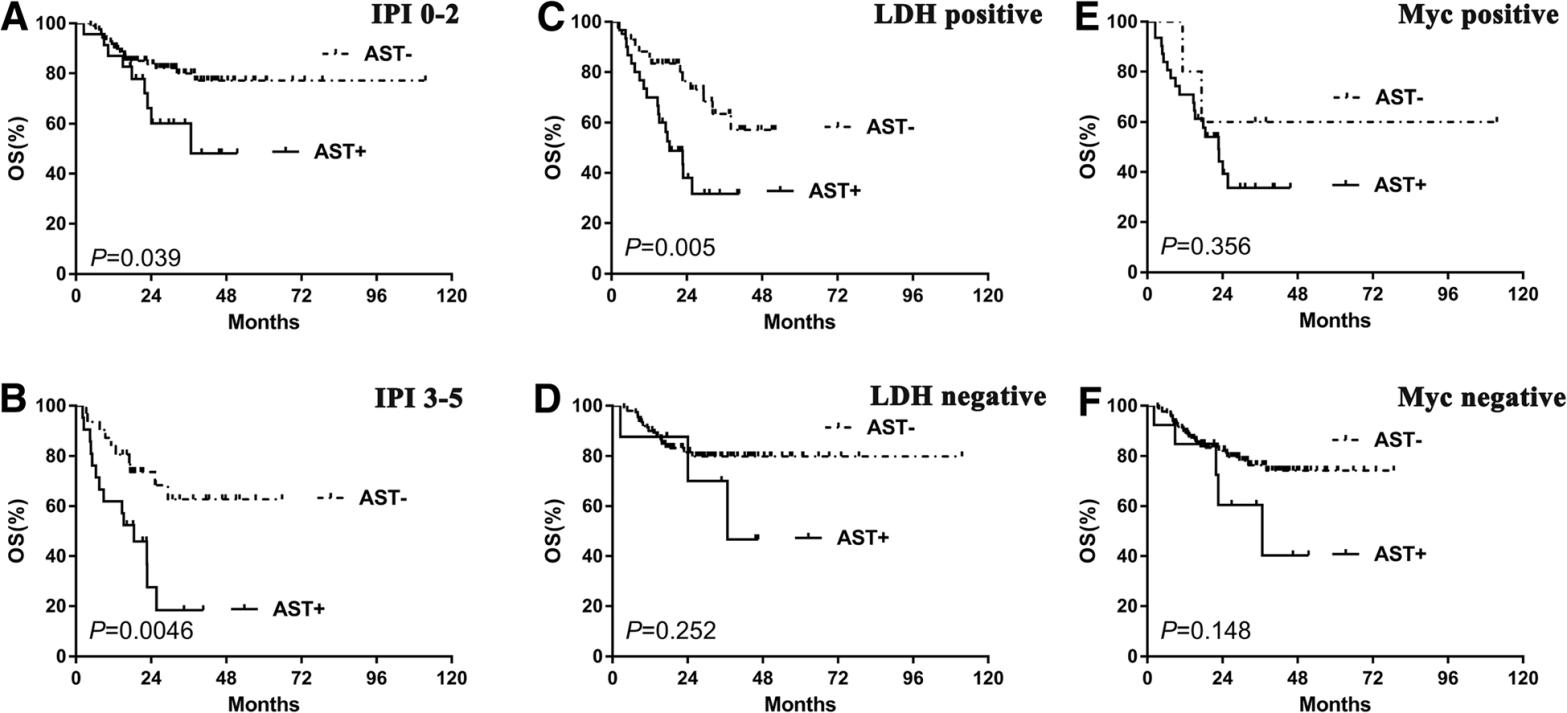
Subgoup analysis with different clinicopathological factors. Patients of higher AST level had superior prognostic value in LDH positive group (**c**). Patients of higher AST level showed poorer outcome regardless of IPI risk (**a-b**). Patients of higher AST had similar OS in different Myc protein status (**e-f**). Abbreviations: AST: aspartate aminotransferase; IPI: International Prognostic Index; LDH: lactate dehydrogenase; OS: overall survival

To extend our findings, survival analysis was performed in our cohort. ALT-positive patients showed similar OS to the negative cases (Fig. 4a). However, AST-positive cases, showed significantly decreased OS compared with the negative ones (2-year OS, 49.2% vs. 81.1%, *P* < 0.0001) (Fig. 4b). Other clinical and pathological factors were also associated with poor survival, including B symptoms, non-GCB subtype, advanced Ann Arbor stage, poor PS, elevated LDH level, ENI ≥ 2, and high-risk IPI (Fig. 4c-h). In subgroup analysis, patients with higher AST levels showed poorer outcomes in patients without B symptoms ((Fig. 5a) and LDH positive groups (Fig. 6c). Higher AST levels showed poorer survival in patients of different ENI (both ENI < 2 and ENI ≥ 2 groups) (Fig. 5c-d), COO (both GCB and non-GCB groups) (Fig. 5e-f) and IPI risk showed (both IPI 0–2 and IPI 3–5 groups) (Fig. 6a, b). Patients of different AST level had similar OS with either Myc protein positive or negative group (Fig. 6e-f).

## Discussion

It has been reported that a Hepatitis B virus (HBV) infection can cause long term necro-inflammatory liver damage, resulting in lower deactivation of estrogen, which is a dominant risk factor for breast cancer [9]. Furthermore, HBV infection has been associated with a greater risk of developing NHL, especially DLBCL, follicular lymphoma, and T cell lymphoma [1]. In addition, HBsAg-positive DLBCL patients have been shown to usually have significantly worse outcomes than HBsAg-negative patients [3]. However, a recent study has indicated that liver impairment and not the viral hepatitis status affects the outcome of DLBCL patients [10]. In the present study, we evaluated the prognostic value of two hepatic enzymes (ALT and AST), routinely assessed in liver function tests, in DLBCL patients who had no evidence of prior HBV infection. The AST level, obtained as a part of a pretreatment liver function test, is a novel prognostic factor in DLBCL patients. This is the first time that the prognostic value of the serum AST level in DLBCL patients has been discussed in published literature. We provide evidence that a higher pretreatment AST level is associated with poorer OS. Furthermore, a higher AST level at diagnosis is significantly related to high-risk clinical features in patients who received R-CHOP or similar chemotherapy regimens. Higher AST level had superior prognostic values in patients of “A symptoms” and LDH positive groups by subgroup analysis.

Levels of ALT and AST are particularly high in the liver. In China, like in many other countries, ALT and AST are usually tested in the same panel [11]. Hypoxia, trauma, ischemia, damage of cell membrane integrity, and function impairment can cause increased cell permeability, mitochondrial swelling, and cell rupture, which in turn cause ALT and AST to be released into the bloodstream. Consequently, the serum AST and ALT levels are closely related to the severity of liver injury [6, 12]. Furthermore, AST is also present in hepatocyte mitochondria and plays a significant role in numerous tissues [11].

Beside normal cells, malignant cells can also generate AST and it has been increasingly recognized that AST plays an important role in carcinogenesis [4, 6, 13, 14]. Studies have demonstrated that a higher AST level is significantly associated with an unfavorable prognosis in numerous cancers, such as hepatocellular and renal cell carcinoma as well as colonic, pancreatic, NSCLC, and breast cancer [6, 15–18]. However, the prognostic value of serum AST levels in patients with malignant lymphoma, especially DLBCL, is still unknown.

This study demonstrates that a high AST level is associated with high-risk clinical features. We must acknowledge that a high AST level was more common in the non-GCB subtype in our study; however, the expression of AST was similar between the GCB and ABC cell lines. This difference might be explained by different sorting techniques and sample sources. The survival analysis presented in this study showed a high AST level predicted a decreased OS in DLBCL patients. However, the prognostic value of AST levels in literature is controversial. Similarly to our study, Kiba et al. have shown that the prognosis of patients with multiple myeloma with a high AST level is worse than the prognosis of patients with a low AST level. [14]. Further research has also suggested that blood-based serum AST is a useful outcome prediction tool in hepatocellular carcinoma [19]. However, Chen and his colleagues have shown that elevated AST levels are significantly associated with longer relapse free survival and OS in NSCLC patients [6].

The underlying relationship between AST and cancer activity is unclear. Glycolysis, which produces ATP and anabolic precursors, is necessary for cancer cells to survive, grow, and invade [20–22]. Some studies have shown that malignant proliferating cells can also obtain energy through glutamine metabolism which is catalyzed by AST [20, 21, 23]. In vitro, oxamate, which targets AST, can inhibit the proliferation of transformed breast adenocarcinoma cells [17]. Thus, high AST levels might in turn promote cell proliferation. In addition, mice with hepatocellular carcinoma (HCC) and liver damage with a high AST level treated with chemotherapy exhibit decreased MYC levels, which can significantly decrease the level of AST and suggest liver function recovery [24]. In our study, we also show that a higher AST level in DLBCL patients was frequently accompanied by higher Myc protein expression. Therefore, we suggest that MYC and AST have a synergetic effect in tumorigenesis, which could indirectly indicate that a higher level of AST is a predictor of poor outcome in DLBCL patients.

Witjes et al. have shown a high AST level and viral load are two independent factors associated with poor survival in HCC and that a high AST level in HCC patients is also associated with a higher HBV DNA [19]. Studies have also confirmed a strong association between HBV infection and DLBCL occurrence [25]. In China, about 13.8% of DLBCL cases are HBV-positive and show unique advanced clinical features [26]. HBV carriers show significantly increased levels of AST. In our study, we noticed a high HBsAg level was associated with an elevated AST level, which indicated dismal outcomes. However, none of these patients with high HBsAg levels had increased HBV DNA copy numbers, which indicated that the raised AST levels were more likely to be caused by lymphoma activity and not by HBV replication. Together, our findings suggest that high AST levels in de novo DLBCL patients are associated with unfavorable prognosis when treated with normal first-line chemotherapy. Thus, more intensive first-line regimens might improve the outcome of these patients.

## Conclusions

This study is limited due to its retrospective and small-scale design; further prospective studies with more patients are required. Nonetheless, our study indicates that a pretreatment AST level is associated with many unfavorable clinical characteristics and is an unfavorable prognostic factor for OS in DLBCL patients treated with normal first-line chemotherapy regimens. We suggest more intensive therapies might overcome the unfavorable outcome of these patients.

## Additional files

Additional file 1: **Figure S1.** Dichotomy of AST level showed a high prognosis value according to X-tile. The optimal cutoff value was 33.3 U/L for AST. (TIF 1208 kb)

Additional file 2: **Figure S2.** Trichotomy of AST level showed a low prognosis value according to X-tile. No optimal cutoff value was observed. (TIF 1213 kb)

## Data Availability

The datasets generated and/or analyzed during the current study are available from the corresponding author on reasonable request.

## Abbreviations

ABC: Activated B-cell type
ALT: Alanine aminotransaminase
AST: Aspartate aminotransferase
COO: Cells of origin
DLBCL: Diffuse large B-cell lymphoma
ECOG PS: Performance status of Eastern Cooperative Oncology Group
ENI: Extra nodal involvement
GCB: Germinal center B-cell type
GEO: Gene expression omnibus
HBsAg: HBV surface antigen
HBV: Hepatitis B virus
HCC: Hepatocellular carcinoma
IPI: International Prognostic Index
LDH: Lactate dehydrogenase
NHL: Non-Hodgkin lymphoma
OS: Overall survival
R-CHOP: Rituximab plus cyclophosphamide, doxorubicin, vincristine, prednisone
