# Supplementary figures and images for: Prognostic significance of serum aspartic transaminase in diffuse large B-cell lymphoma
Source: BMC Cancer. 2019 Jun 8;19:553. doi: 10.1186/s12885-019-5758-2 (PMC6556027; doi:10.1186/s12885-019-5758-2)

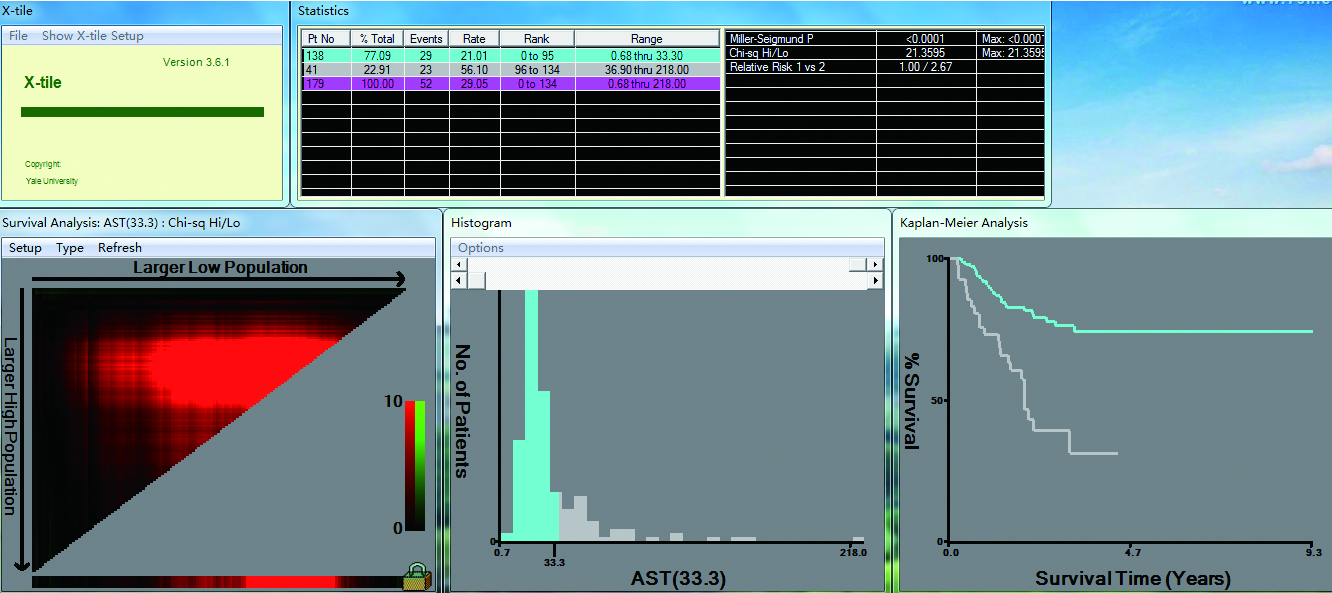

Supplement: Supplementary file 1 — Figure S1. Dichotomy of AST level showed a high prognosis value according to X-tile. The optimal cutoff value was 33.3 U/L for AST. (TIF 1208 kb) [file 12885_2019_5758_MOESM1_ESM.tif]

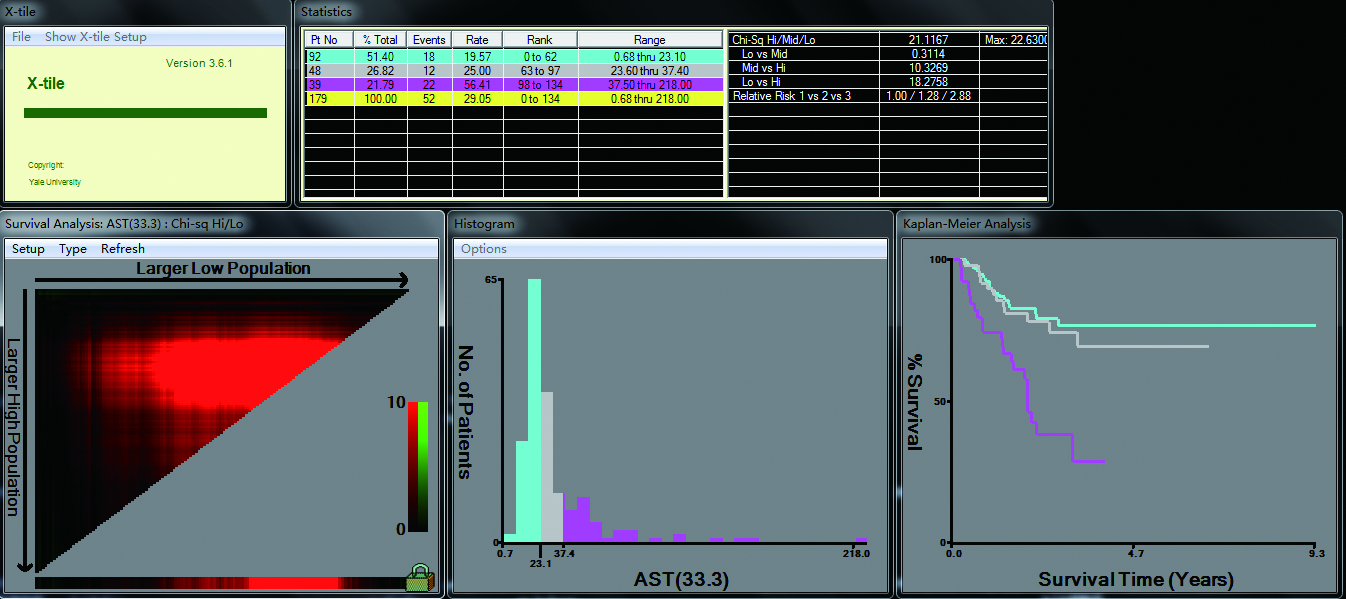

Supplement: Supplementary file 2 — Figure S2. Trichotomy of AST level showed a low prognosis value according to X-tile. No optimal cutoff value was observed. (TIF 1213 kb) [file 12885_2019_5758_MOESM2_ESM.tif]
